# Massive mining of publicly available RNA-seq data from human and mouse

**DOI:** 10.1038/s41467-018-03751-6

**Published:** 2018-04-10

**Authors:** Alexander Lachmann, Denis Torre, Alexandra B. Keenan, Kathleen M. Jagodnik, Hoyjin J. Lee, Lily Wang, Moshe C. Silverstein, Avi Ma’ayan

**Affiliations:** Department of Pharmacological Sciences; Mount Sinai Center for Bioinformatics; Big Data to Knowledge, Library of Integrated Network-based Cellular Signatures, Data Coordination and Integration Center (BD2K-LINCS DCIC); Knowledge Management Center for Illuminating the Druggable Genome (KMC-IDG), Icahn School of Medicine at Mount Sinai, One Gustave L. Levy Place, Box 1603, New York, NY 10029 USA

## Abstract

RNA sequencing (RNA-seq) is the leading technology for genome-wide transcript quantification. However, publicly available RNA-seq data is currently provided mostly in raw form, a significant barrier for global and integrative retrospective analyses. ARCHS4 is a web resource that makes the majority of published RNA-seq data from human and mouse available at the gene and transcript levels. For developing ARCHS4, available FASTQ files from RNA-seq experiments from the Gene Expression Omnibus (GEO) were aligned using a cloud-based infrastructure. In total 187,946 samples are accessible through ARCHS4 with 103,083 mouse and 84,863 human. Additionally, the ARCHS4 web interface provides intuitive exploration of the processed data through querying tools, interactive visualization, and gene pages that provide average expression across cell lines and tissues, top co-expressed genes for each gene, and predicted biological functions and protein–protein interactions for each gene based on prior knowledge combined with co-expression.

## Abstract (web summary)

Publicly available RNA-seq data is provided mostly in raw form, resulting in a barrier for integrative analyses. Here, Lachmann et al. develop a high-throughput processing infrastructure and search database (ARCHS4) that provides processed RNA-seq data for 187,946 publicly available mouse and human samples to support exploration and reuse.

## Introduction

**Fig. 1:**
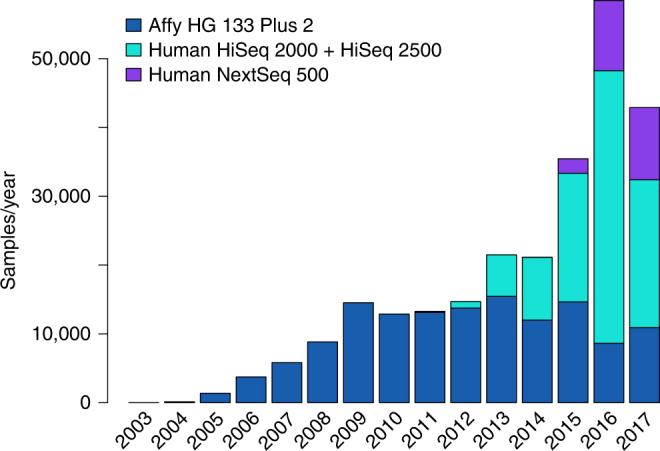
Publicly available RNA-seq samples currently available at GEO/SRA for human and mouse compared to available samples collected with the popular Affymetrix HG U133 Plus 2 platform

The completion of the Human Genome Project^1^ enabled the quantification of mRNA expression at the genome-wide scale, initially with cDNA microarray technology^2^, but now more commonly via RNA-sequencing (RNA-seq) (Fig. 1). RNA-seq is replacing cDNA microarrays as the dominant technology because it offers reduced cost, increased sensitivity, ability to quantify splice variants and perform mutation analyses, improved quantification at the transcript level, identification of novel transcripts, and improved reproducibility^3^. Genome-wide gene expression data from thousands of studies have been accumulating and made available for exploration and reuse through public repositories such as the Gene Expression Omnibus (GEO)^4^ and ArrayExpress^5^. Since the late 1990s software for the analysis of cDNA microarray data has matured toward established community accepted computational procedures, whereas analyses methods to process RNA-seq data are still actively refined and developed.

The quality of RNA-seq data depends on the sequencing depth whereby more reads per sample can reduce technical noise. Modern sequencing platforms such as Illumina HiSeq produce tens of millions of paired-end reads of up to 150 base pairs in length per sample. The raw reads are aligned to a reference genome by mapping reads to known gene sequences. The alignment step is computationally demanding, and the various alignment algorithms implemented in software packages are continually improving^6–12^. Bowtie^6^ is one of the first alignment methods that gained wide spread popularity. More efficient solutions were later implemented by improving memory utilization with faster execution time. One of the currently leading alignment methods, Spliced Transcripts Alignment to a Reference (STAR)^8^, can map more than 200 million reads per hour. As a trade-off for increased computational speed, STAR requires heavy memory consumption, particularly for large genomes such as human or mouse. For mammalian genomes, STAR requires more than 30 GB of random access memory (RAM). This requirement limits its application to high performance computing (HPC) platforms. This introduces a barrier for the typical experimental biologist who generates the data. Additionally, knowledge in programming and a series of choices in regards to the alignment software parameter settings, are commonly required to covert raw reads to a quantified expression matrix of processed RNA-seq data.

Retrospective analyses of large collections of previously published RNA-seq data can illuminate new biology and accelerate drug discovery^13^. However, many post hoc studies rely on large data sets that are interoperable with data analysis workflows whereby gene expression data is provided in processed form. For example, the Genotype-Tissue Expression project (GTEx)^14^ and The Cancer Genome Atlas (TCGA)^15^ RNA-seq data sets are frequently reused in post hoc projects mainly because such data are provided in a useful processed format. GTEx currently contains 9662 RNA-seq samples from 53 human tissues collected from over 250 individuals, whereas TCGA contains at least 11,077 RNA-seq samples created from a diverse collection of tumors.

**Fig. 2:**
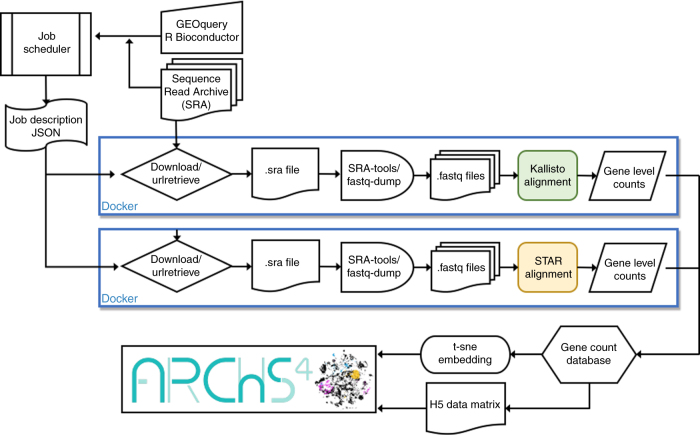
Schematic illustration of the ARCHS4 cloud-based alignment pipeline workflow. A job scheduler instructs Dockerized alignment instances that are processing FASTQ files from the SRA database in parallel. The pipeline supports the STAR and Kallisto aligners. The final results are sent to a database for post-processing. Dimensionality reduction for data visualization is calculated with t-SNE, and all counts are additionally stored in a H5 data matrix. The .sra file extension is the native file format for files from the SRA database

Recent efforts attempted to simplify the access to gene expression data collected via RNA-seq to create more unified resources from fragmented repositories^16–20^. Currently, as of February 2018, there are 187,946 RNA-seq samples, collected from human or mouse cells and tissues, that are accessible from the Gene Expression Omnibus (GEO) and the Sequence Read Archive (SRA), making this resource the most comprehensive repository for RNA-seq data collected from mammals. This large collection of samples from diverse institutions, laboratories, studies, and projects is comprehensive, but not homogeneous compared with RNA-seq data collected for large projects such as GTEx and TCGA. The data within GEO/SRA is provided mostly in raw sequence form. While some studies provide aligned reads files, these are few and processed non-uniformly. This shortcoming makes it difficult to query and integrate this data at a global scale. To bridge the gap that currently exists between RNA-seq data generation and RNA-seq data processing, we developed the resource all RNA-seq and ChIP-seq sample and signature search (ARCHS4). The ARCHS4 pipeline (Fig. 2) processes RNA-seq data from GEO/SRA to support retrospective data analyses and reuse. ARCHS4 caters to users with different levels of computational expertise and can be employed for many post hoc analyses and projects. The goal is to provide users with direct access to the data through a web-based user interface, while implementing a scalable and cost-effective solution for the raw data processing task. The usefulness of ARCHS4 is exemplified through case studies that show how the data assembled can be applied to predict gene function and protein–protein interactions (PPI).

## Results

### The ARCHS4 website

**Fig. 3:**
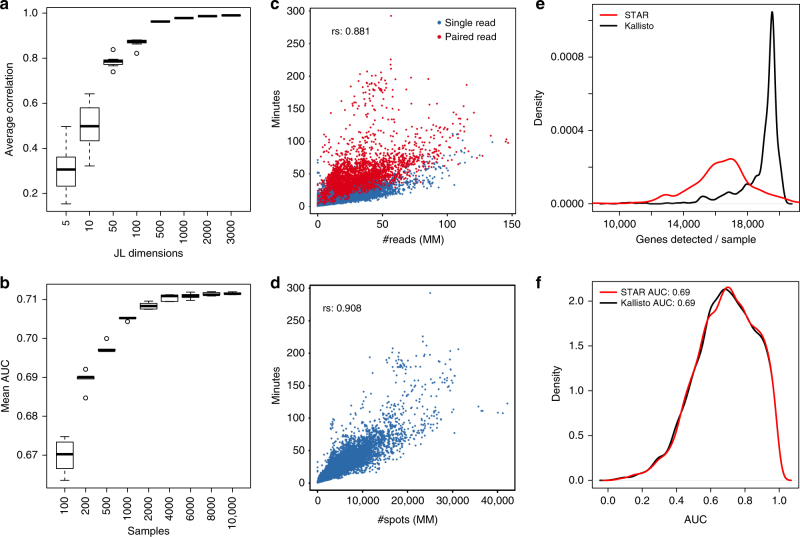
Dimensionality reduction and processing time evaluation. **a** Average correlation between samples before and after applying the Johnson–Lindenstrauss dimensionality reduction. The original gene expression matrix is reduced from 34,198 genes/dimensions to smaller sets of JL dimensions. For each number of JL dimensions, the procedure was repeated 10 times to obtain variances. **b** Mean AUC for predicting GO biological processes using the ARCHS4 mouse co-expression data created from different size sets of randomly selected samples. Whiskers in plots **a** and **b** represent one standard deviation from the mean. **c** Processing time per million reads for single read and paired-end read RNA-seq for the Kallisto processing container. **d** Elapsed time per million (MM) spots/nucleotides for completing the processing of paired read FASTQ files with the Dockerized Kallisto processing container; rs in **c** and **d** are the *r*^2^ correlation coefficient linear fit. **e** Distribution of the number of detected genes for pipelines that utilize the Kallisto vs. STAR aligners across 1708 randomly selected and processed human RNA-seq samples. **f** Distribution of AUCs for predicting gene set membership for GO biological processes from co-expression matrices derived from the same set of 1708 human RNA-seq samples processed by STAR or Kallisto aligners

The ARCHS4 website supports multiple complementary ways to access the processed RNA-seq gene expression data. For programmatic access, the download section provides access to all the processed data for human and mouse in H5 format. The H5 files contain extensive metadata retrieved from GEO. This metadata can be queried to extract samples of interest by keywords. Additionally, programmatic access to ARCHS4 supports exploration of gene expression matrices through search functions. The ARCHS4 website visualizes all the processed samples, and alternatively all human or mouse genes, based on their co-expression similarity, as interactive 3D t-Distributed Stochastic Neighbor Embedding (t-SNE) plots. In the samples view, all samples can be searched by metadata terms. ARCHS4 performs text searches of the GEO metadata to retrieve samples by matching terms. For example, searching Pancreatic Islet in the human context, will return 1829 samples from 10 independent GEO series. After the search is complete, the samples are highlighted and an auto-generated R script is provided for downloading the set of highlighted samples. Executing the R script builds a local expression matrix in tab-separated values format with the samples as columns and the genes as the rows. The signature search in ARCHS4 enables searching samples at the data level, matching high and low expressed genes from input sets of high/low expressed genes across all ARCHS4 processed samples. Signature similarity is approximated via the Johnson–Lindenstrauss (JL)^21^ transformed gene expression space that preserves the global structure of the data while reducing its dimensionality (Fig. 3a, b). Under the enrichment search tab, samples can be selected by annotated prior knowledge gene sets. Gene set libraries from which annotated gene sets are currently derived are: ChEA^22^, ENCODE^23^, KEA^24^, Gene Ontology (GO)^25^, KEGG PATHWAY database^26^, and MGI mammalian phenotypes^27^.

The ARCHS4 three-dimensional viewer also supports manual lasso selection of samples through a snipping tool. The colors used to highlight selected samples can be changed by the user. The genes view of ARCHS4 provides the same manual selection feature as with the samples view. Selected gene lists can be downloaded directly from ARCHS4, or submitted for gene list enrichment analysis with Enrichr^28,29^. Additionally, individual genes can be queried to locate an ARCHS4-dedicated gene-landing page. These single gene landing pages contain predicted biological functions based on correlations with genes assigned to GO categories; predicted upstream transcription factors based on correlation with identified targets as determined by ChIP-seq data from the ChEA and ENCODE gene set libraries; predicted knockout mouse phenotypes based on annotated MGI mammalian phenotypes; predicted human phenotypes based on co-expression correlation with genes that have assigned human phenotypes in the Human Phenotype Ontology^30^; predicted upstream protein kinases based on known kinase-substrates from KEA; and membership in pathways based on co-expression with pathways from KEGG. The single gene landing pages also list the top 100 most co-expressed genes for each individual gene. Additionally, for 53 distinct tissues and 67 cell lines, expression levels are visualized for each gene. These are visualized as two hierarchical trees with tissues and cells grouped by system and organ.

In addition, ARCHS4 processed data can be accessed via the ARCHS4 Chrome extension, which is freely available from the Chrome Web Store. The Chrome extension detects GEO series landing pages and then inserts a Series Matrix File (SMF) for download for each series that has been processed by the ARCHS4 pipeline. Each SMF contains read counts for all available samples in the series. The sample expression is also visualized as a heatmap using the Clustergrammer plugin^31^. Clustergrammer loads JSON files containing the *z*-score normalized gene expression of the top 500 most variable genes across the series and embeds the interactive heatmap directly into the GEO series landing page.

### RNA-seq alignment pipeline speed and cost

The ARCHS4 pipeline (Fig. 2) speed is measured by the elapsed time from job submission until completion for 31,825 samples. The timed process includes: downloading the SRA file, extracting the FASTQ file from the SRA data format, alignment to the reference genome, mapping the transcript counts to the gene level, and writing the final result to the database. Processing time of a single FASTQ file takes on average ~11 min. This benchmark is applied using Amazon EC2 on-demand m4.large instances with 8 GB of memory and 2 vCPUs, running with 200 GB of hard-drive storage. Each instance can run 2 Dockerized alignment pipeline containers in parallel. At the time of the benchmark, the cost of the on-demand m4.large instances was $0.1/h. This results in an average compute cost for one processed SRA file to be $0.00982. For samples with at least 1,000,000 aligned reads and paired reads with 200 bp, the alignment cost was $0.973 per billion reads or $0.00486 per billion base pairs. For these specific samples, this averages to $0.025 per sample. Most samples, however, have unpaired reads and lower read counts, resulting in the lower cost of less than a cent. The alignment time correlates with the number of reads (Spearman’s correlation coefficient *r* = 0.881), and the processing time increases linearly with the number of spots aligned with some variance (*r* = 0.901, paired reads) due to performance differences between cloud computing instances (Fig. 3c, d). Paired-end read RNA-seq experiments require more time during the alignment process due to the increased number of spots that have to be processed. The ARCHS4 pipeline is, to our knowledge, the most cost-effective cloud-based RNA-seq alignment infrastructure published to date.

### STAR vs. Kallisto comparison

To achieve its fast and cost-effective solution, ARCHS4 utilizes the Kallisto aligner^9^. However, it is not clear whether the improved speed and cost provided by Kallisto comes with a cost of drop in the output quality. To benchmark Kallisto against other aligners, a subset of 1708 human samples processed by ARCHS4 were also aligned with STAR^8^. While Kallisto and STAR return similar gene expression profiles, there are profound differences between the output produced by the two methods. In general, Kallisto detects more genes than STAR (Fig. 3e, f). The average Pearson correlation of the *z*-score transformed samples between the Kallisto and STAR outputs is 0.77. However, the number of detected genes does not directly translate to a qualitative advantage of Kallisto over STAR. To test the quality of the generated gene expression matrices and their gene correlation structure, we tested the ability of the processed data sets by STAR or Kallisto to predict GO biological processes for single genes, as described in detail in the Methods section. The quality of the predictions is almost identical for the two compared data sets with an average area under the curve (AUC) of 0.69 for predictions made by processed data generated by the two separate methods (Fig. 3f).

### Comparison to existing RNA-seq pipelines and resources

**Fig. 4:**
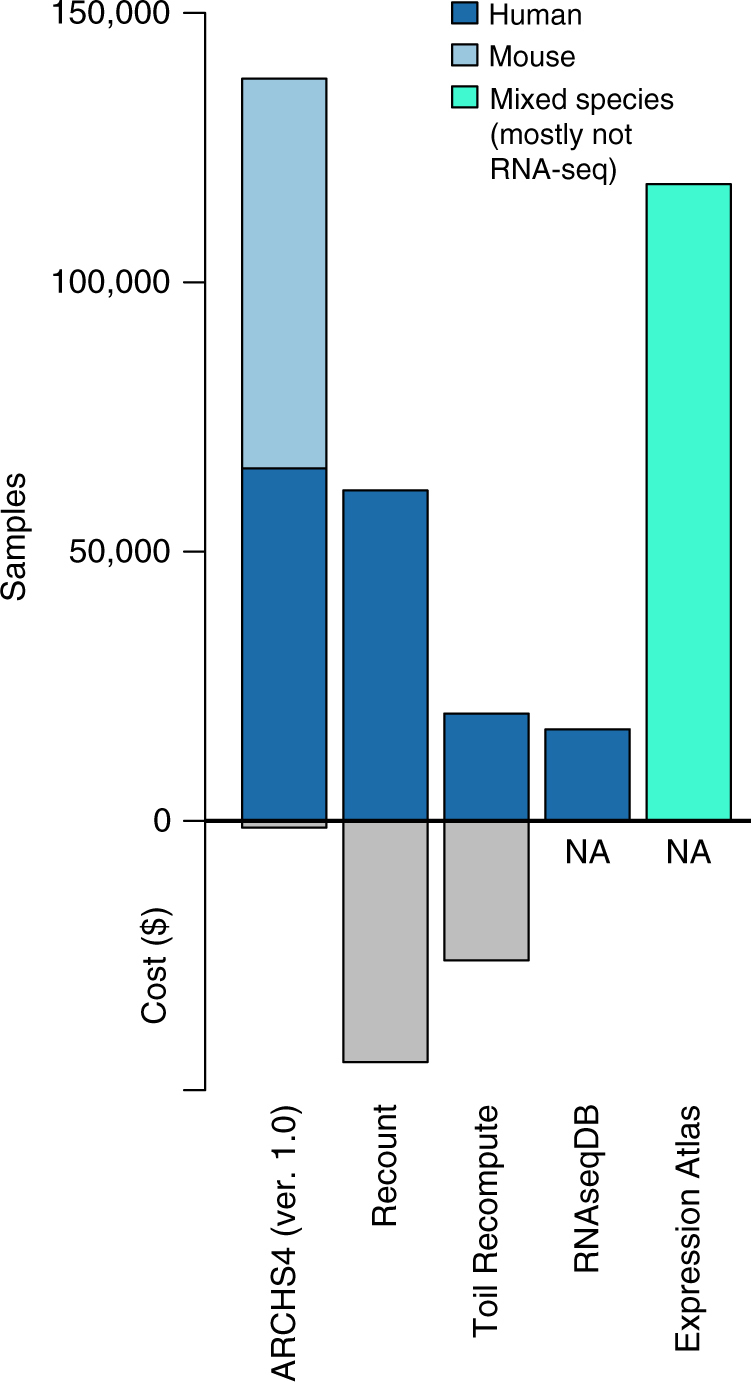
Total available samples from large-scale re-processing RNA-seq resources and the total estimated cost of processing raw samples to gene/transcript counts

Multiple efforts attempted to uniformly reprocess large collections of RNA-seq data^16–20^. Table 1 provides an overview comparison of several resources with respect to size, cost per sample, and other attributes. The total sample size and cost for data processing are visualized in Fig. 4. Even though ARCHS4 contains more than double the number of RNA-seq samples than other resources, the estimated costs compared with Recount and Toil Recompute is an order of magnitude lower with $1745, $44,785, and $25,910, respectively. All approaches rely on the use of either private or public high-performance computer clusters. Toil Recompute^16^ was applied to re-compute the transcript level counts of 19,931 RNA-seq samples. The UCSC pipeline architecture was run on an Amazon Web Services (AWS) cluster and averaged $1.30 per sample. The data set contains 11,194 samples from TCGA, 8002 from GTEx, and 734 additional samples. The processed data is made available through a web interface called the Xena browser. Expression Atlas^17^ provides processed RNA-seq and microarray gene expression data for multiple species. Expression data is processed by a pipeline named iRAP^18^. The total number of assays in Expression Atlas is 118,209 from 3035 experiments. From these, only 565 are RNA-seq. All data is reported at the gene level and is accessible as a bulk zip download.

**Table 1:** Comparison of processed RNA-seq resources

RNA-seq resource	ARCHS4	Recount	Toil Recompute	RNAseqDB	Expression Atlas
Human samples	84,863	61,350	19,931	>17,000	NA
Mouse samples	103,083	0	0	0	NA
Total samples	187,946	61,350	19,931	> 17,000	118,209^a^
Cost per sample	< $0.01	$0.73	$1.30	NA	NA
Gene level	✓	✓	✗	✗	✓
Transcript level	✓	✓	✓	✓	✗
Alignment-free quantification	✓	✗	✗	✗	✗
API support	✓	✓	✗	✗	✗
Chrome extension	✓	✗	✗	✗	✗
Data query	✓	✗	✗	✗	✓
Enrichment	✓	✗	✗	✗	✓

^a^Mostly not RNA-seq, only ~500 samples are from RNA-seq

The number of samples covered by ARCHS4, Recount, Toil Recompute, RNAseqDB, and Expression Atlas as well as features of the web resource are listed and compared

The Recount project^19^ performed sequence alignment with a pipeline termed Rail-RNA. The reported cost per sample is $0.72. The data in Recount contains 9662 samples from GTEx, 11,350 from TCGA, and ~50,000 human SRA samples. The data is available as bulk download or through an R package. Expression is reported at the gene and transcript levels. The RNAseqDB^20^ also contains all GTEx and TCGA processed samples. The alignment for RNAseqDB was performed on an internal cluster and no cost analysis is available. The data is provided as bulk download files with FPKM normalized transcripts. The data is deposited in a GitHub repository. In contrast with ARCHS4, the reported cost for all similar efforts is about two orders of magnitude more expensive for processing a sample. Cost per sample is a critical factor in processing RNA-seq data because of the rapid growth in data production. Compute cost for ARCHS4 is almost negligible compared to the cost of comparable efforts. The number of samples already available from the ARCHS4 resource is by far the largest collection of processed RNA-seq to date, and the low-cost pipeline enables a rolling update as more samples become available. In contrast with other resources, ARCHS4 provides multiple methods for data accessibility. While Recount and Expression Atlas support programmatic access through R packages, only the Expression Atlas supports enrichment analysis on signatures derived from the expression profiles. A unique feature of ARCHS4 is the real-time data and metadata querying support that allows the identification and selection of relevant subsets of samples.

### Read quality across institutions

**Fig. 5:**
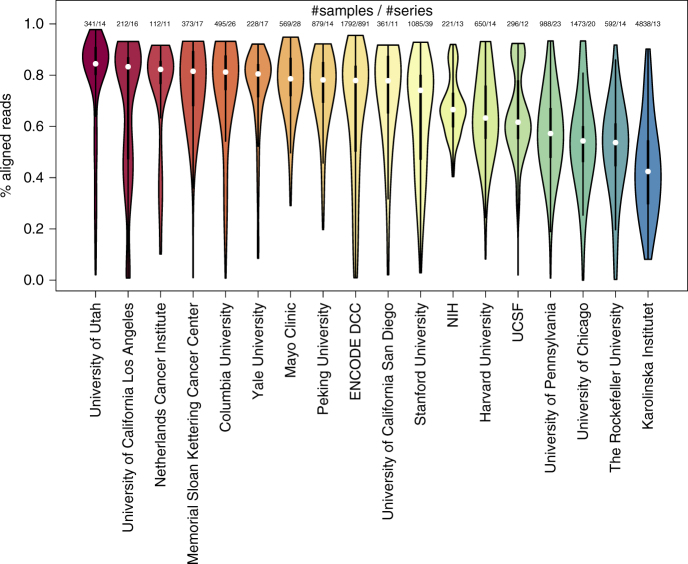
Distribution of the percentage of aligned reads from human RNA-seq samples that are successfully aligned with Kallisto by institution as it is reported within GEO submission pages. The selected institutions that are shown, have processed at least 100 samples from more than 10 different gene expression series. Colors represent alignment quality (red-high; blue-low)

The percentage of aligned reads over total reads for each FASTQ file varies significantly across labs, projects, and sequencing cores due to various reasons. Since each sample from GEO/SRA is annotated with the producers of the data, the percent of aligned reads by institution can be plotted (Fig. 5). The 34 institutions that so far produced more than 100 unique samples from more than 20 gene expression series of RNA-seq samples available on the GEO/SRA database show that the highest percentage of successful aligned reads is by the University of Minnesota with a median of 87%. The 429 samples that originated from the Jackson Laboratory come from 23 distinct gene expression series. It should be noted that observed differences in the fraction of aligned reads is not necessarily an indicator of the performance of the sequencing core within an institution but can be attributed to the quality of the samples. For example, samples from formalin-fixated tissues will suffer from RNA degradation which will result in lower percent of aligned reads. In addition, it should also be noted that in general investigators from most research institutions frequently use various external sequencing core services. On average, 63% of reads were aligned across all 84,863 of the processed human RNA-seq samples, whereas 59% of all mouse RNA-seq reads from 103,083 samples were aligned to matching genes.

### Prediction of biological functions and protein interactions

**Fig. 6:**
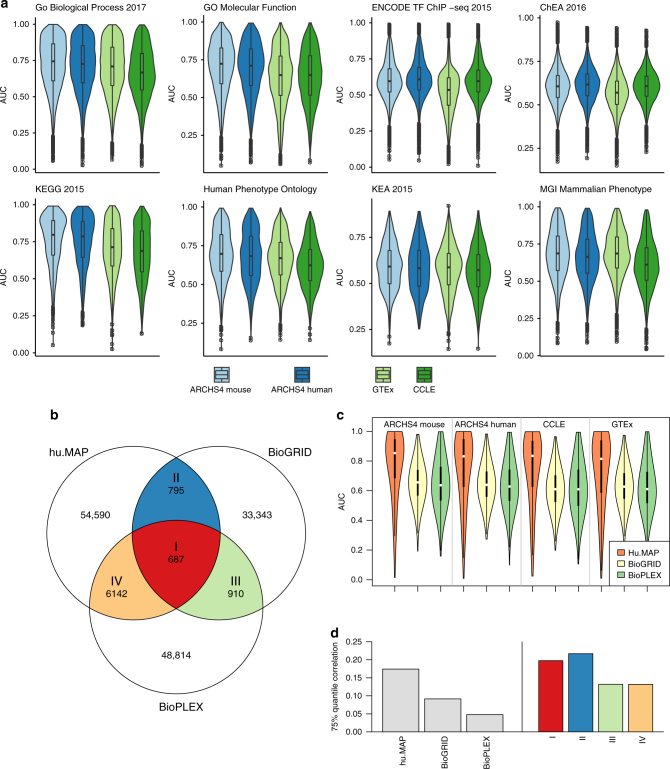
Prediction of biological function and protein–protein interactions. **a** The distribution of AUC for gene set membership prediction of gene annotations from eight gene set libraries with co-expression data created from ARCHS4 mouse, ARCHS4 human, GTEx, and CCLE. The gene set libraries used to train and evaluate the predictions are ChEA, ENCODE, GO Biological Process, GO Molecular Function, KEA, KEGG Pathways, Human Phenotype Ontology, and MGI Mammalian Phenotype Level 4. These libraries were obtained from the Enrichr collection of libraries. **b** Venn diagram showing the intersection of edges between three PPI databases hu.MAP, BioGRID, and BioPLEX. **c** Distribution of AUC for protein–protein interaction prediction from gene co-expression data created in the same way from ARCHS4 mouse, ARCHS4 human, CCLE, and GTEx. **d** Bar plot of the pairwise correlation between genes with reported protein–protein interactions for the three PPI networks hu.MAP, BioGRID, and BioPLEX in ARCHS4 mouse expression. The right tail of the gene pair correlation distribution is shown by the 75% quantile. On the right, the bars represent the percent overlap of predicted interactions for the matching intersections from the Venn diagram plotted in **b**

Gene function and PPI can be potentially predicted using co-expression data, whereas the data that is processed for ARCHS4 provides a rich resource for generating gene co-expression correlations. Evaluating the quality of co-expression correlation networks to predict protein interactions and biological functions can also provide an unbiased benchmark to compare the ARCHS4 resource with other major RNA-seq and microarray repositories. The hypothesis is that gene function and protein interactions can be predicted using co-expression data, and this implies that co-expressed genes tend to share their function and physically interact. It means that genes are assigned predicted biological functions when they are highly correlated with a set of genes that are already annotated to have some biological function. Similarly, a gene product is predicted to interact with another protein if the known direct protein interactors for that other protein are highly co-expressed with the gene product protein. We evaluate the human and mouse ARCHS4 data sets by comparing them to co-expression matrices created in the same way from the Cancer Cell Line Encyclopedia (CCLE) and GTEx resources. All gene expression data sets produce, on average, significant ability to predict both biological functions and protein interactions. This suggests that gene expression correlations derived from large-scale expression data sets are predictive of biological function and protein interactions. In almost all the tested categories, the ARCHS4 mouse and human data sets significantly outperformed the predictions made with co-expression data created from the CCLE and GTEx data sets (Table 2). The most accurate predictions for GO biological processes, GO molecular functions, KEGG pathways, Human Phenotype Ontology terms, predicted upstream kinases, and MGI Mammalian Phenotype terms are achieved with the ARCHS4 mouse gene co-expression data followed by the ARCHS4 human data. The co-expression data from GTEx outperforms the co-expression data created from the CCLE for GO biological processes and the phenotype libraries, whereas the predictability of using GTEx data in the same way is lower than CCLE for the upstream regulatory transcription factor predictions. *P*-values are calculated for the ∆ mean between methods. For the ARCHS4 mouse co-expression data, the AUC distributions for predicting gene function are significant across all categories, but most successfully in predicting GO biological processes with median AUC of 0.745, and membership in KEGG pathways with a median AUC of 0.797 (Fig. 6a).

**Table 2:** Comparison of functional prediction for ARCHS4 mouse and human gene expression compared to GTEx and CCLE

Gene set library		ARCHS4 mouse	ARCHS4 human	GTEx	CCLE
Go Biological Process 2017	Median	0.745	0.726	0.709	0.667
	Δ median	0	−0.0186	−0.0356	−0.0773
	*p*-value	1	7.70E−08	1.47E−27	8.50E−124
GO molecular function	Median	0.724	0.71	0.649	0.649
	Δ median	0	−0.0134	−0.0752	−0.0752
	*p*-value	1	0.0174	1.08E−78	7.93E−64
ENCODE TF ChIP-seq 2015	Median	0.596	0.608	0.5349	0.596
	Δ median	0	0.0124	−0.061	0.000271
	*p*-value	1	5.54E−13	0	0.000476
ChEA 2016	Median	0.606	0.617	0.57	0.608
	Δ median	0	0.0104	−0.0363	0.00139
	*p*-value	1	1.95E−17	5.44E−266	0.758
KEGG 2015	Median	0.797	0.786	0.713	0.688
	Δ median	0	−0.0109	−0.0838	−0.109
	*p*-value	1	0.21	5.76E−20	2.56E−35
Human phenotype ontology	Median	0.698	0.683	0.669	0.623
	Δ median	0	−0.0144	−0.0284	−0.0745
	*p*-value	1	0.00251	2.38E−10	6.05E−48
KEA 2015	Median	0.591	0.583	0.587	0.572
	Δ median	0	−0.0088	−0.00439	−0.019
	*p*-value	1	0.431	0.0459	0.00365
MGI mammalian phenotype	Median	0.687	0.6639	0.686	0.612
	Δ median	0	−0.0227	−0.000726	−0.0749
	*p*-value	1	3.83E−08	0.537	9.97E−83

Δ median is the difference in median AUC between ARCHS4 mouse and the other data sets. The significance of difference of the mean is calculated by *t*-test for observed AUC distributions.

While predicting protein function with co-expression data has been attempted successfully by many before, it is less established whether co-expression data can be used to also predict PPI. A similar strategy was employed to predict PPI using prior knowledge about PPI. PPI data is fetched from three PPI resources: hu.MAP^32^, BioGRID^33^, and BioPLEX^34^. These three PPI resources are unique. After filtering, the PPI from BioGRID are the composition of interactions from thousands of publications that report only few interactions. The PPI from BioPLEX are bait-prey interactions from a massive mass-spectrometry experiment. The hu.MAP PPI consist of data from three mass-spectrometry experiments integrated with sophisticated computational methods. hu.MAP also considers prey-prey interactions to boost interaction confidence. Importantly, none of these three resources utilize knowledge from mRNA co-expression data to confirm PPI. The overlap of shared interactions between the three PPI networks is relatively low, with hu.MAP and BioPLEX sharing more than 10% of their interactions (Fig. 6b). This is likely because a part of BioPLEX is contained within hu.MAP. Predicting PPI using knowledge from these three PPI resources, the ARCHS4 mouse co-expression data is the most predictive, with median AUCs of 0.85, 0.66 and 0.64 for hu.MAP, BioGRID, and BioPLEX respectively (Fig. 6c).

The fact that PPI from hu.MAP can be predicted at a much higher accuracy compared to the other two networks may suggest that PPI within hu.MAP are more correct. The 75% quantile of interaction correlation in hu.MAP is 0.174 compared to BioGRID (0.0915) and BioPLEX (0.0478); whereas the intersections between the PPI networks (I, II, III, IV) tend to have a higher 75% quantile of correlations, with 0.198, 0.217, 0.131 and 0.132 suggesting that aggregating evidence from experiments that detect PPI, is most likely to boost confidence of real interactions (Fig. 6d). This suggestion further supports that mRNA co-expression data can be used to predict PPI. The predicted PPI and predicted biological functions provide a plethora of computational hypotheses that could be further validated experimentally.

## Discussion

The ARCHS4 resource of processed RNA-seq data is created by systematically processing publicly available raw FASTQ samples from GEO/SRA. This resource can facilitate rapid progress of retrospective post hoc focal and global analyses. The ARCHS4 data processing pipeline employs a modular Dockerized software infrastructure that can align RNA-seq samples at an average cost of less than a cent (US $0.01). To our knowledge, this is an improvement of more than an order of magnitude over previously published solutions. The automation of the pipeline enables constant updating of the data repository by regular inclusion of newly published gene expression samples. The pipeline is open source and available on GitHub so it can be continually enhanced and adopted by the community for other projects. The pipeline uses Kallisto as the main alignment algorithm that was demonstrated through an unbiased benchmark to perform as well as, or even better than, another leading aligner, STAR. We compared the ARCHS4 co-expression data with co-expression data we created from other existing gene expression resources, namely GTEx and CCLE, and demonstrated how co-expression data from ARCHS4 is more effective in predicting biological functions and protein interactions. This could be because the data from ARCHS4 is more diverse. The fact that the data within ARCHS4 is from many sources has its disadvantages. These include batch effects and quality control inconsistencies. Standard batch effect removal methods are not applicable to the entire ARCHS4 data but may be useful for improving the analysis of segments of ARCHS4 data. The ARCHS4 web application and Chrome extension enable users to access and query the ARCHS4 data through both metadata and data searches. For data-driven queries, the unique JL dimensionality reduction method is implemented to maintain pairwise distances and correlations between samples even after reducing the number of dimensions by two orders of magnitude. Reducing the data to a lower dimension facilitates data-driven searches that return results instantly. The gene expression data provided by ARCHS4 is freely accessible for download in the compact HDF5 file format allowing programmatic access. The HDF5 files contain all available metadata information about all samples, but such metadata can be improved by having it follow community standards such as linking it to established identifiers and biological ontologies. The ARCHS4 three-dimensional data viewer lets users gain intuition about the global space of gene expression data from human and mouse at the sample and gene levels. The interface supports interactive data exploration through manual sample selection and highlighting of samples from tissues and cell lines. With the available data, we constructed comprehensive gene landing pages containing information about predicted gene function and PPI, co-expression with other genes, and average expression across cell lines and tissues. For a variety of tissues and cell lines, gene expression distributions are calculated for each gene. Such data can complement tissue and cell line expression resources such as BioGPS^35^ and GTEx^14^ as well as resources that provide accumulated knowledge about genes and proteins such as GeneCards^36^, the Harmonizome^37^, and the NCBI gene database^38^. Overall, the ARCHS4 resource contains comprehensive processed mRNA expression data that can further enable biological discovery toward better understanding of the inner-workings of mammalian cells.

## Methods

### RNA-seq data processing pipeline

The RNA-seq processing pipeline employed for ARCHS4 runs in parallel on the AWS cloud. The core component of the pipeline is the alignment of raw reads to a reference genome. This process is encapsulated in deployable Docker containers^39^ that currently support alignment with two leading fast aligners: STAR^8^ and Kallisto^9^. The memory requirement for a Kallisto Docker image is 4 GB, and for STAR 30 GB. All available SRA files are identified by downloading the GEO series (GSE) and GEO samples (GSM and SRA information) using the GEOquery Bioconductor package^40^. Unprocessed SRA files are entered as jobs into a scheduler database. The job scheduler is a Dockerized web application with APIs that communicate job instructions to worker instances, and for saving the final gene and transcript count files. To allow efficient scaling of computational resources, AWS auto-scaling is utilized in combination with the cluster management console (ECS). For Kallisto instances, a task definition is specified running a Docker image hosted publicly at Docker Hub with 1 vCPU and a 3.9 GB memory limit. The number of desired tasks specifies how many Docker images are to run in parallel. For ARCHS4, we ran 400 Docker instances of Kallisto in parallel due to AWS cap of 200 EC2 instances. The auto-scaling group is set to launch 200 m4.large general compute instances with 2 vCPUs and 8 GB of memory and 200 GB of SSD disk storage. Each instance is capable of running 2 Kallisto Docker instances in parallel. The alignment Docker container, once launched, continuously requests alignment jobs from the job scheduler. The job description contains the SRA file URL and the reference genome. The SRA file is downloaded from the SRA database, while fastq-dump from SRA tools is used to detect single or paired reads file. Then, the SRA file is converted into FASTQ format. In case of a paired read file, the data is split into two FASTQ files. Kallisto or STAR are then used to align the reads against the reference genome. The resulting output is reduced to gene or transcript counts and uploaded through the job scheduler API to the gene and transcript count database. The complete workflow is visualized as a flow chart (Fig. 2). For a subset of 1708 FASTQ files, reads were aligned using STAR. These were selected for a separate project with the aim to generate differential gene expression signatures for single gene, drug/small-molecule, or disease perturbations. The Docker^39^ container maayanlab/awsstar was deployed on a local Mesosphere platform^41^ running on a Mac Pro with 3.7 GHz Quad-Core Intel Xeon E5 and 32 GB of RAM. The supported genomes are Ensembl *Homo sapiens* GRCh38 with the GRCh38.87 annotation file, and Mus Musculus GRCm38 with the GRCm38.88 annotation file.

### Post-processing to make the RNA-seq data accessible

To integrate new samples into the ARCHS4 resource, gene and transcript count files are extracted from the database and saved into HDF5 files^42^. The HDF5 files for human and mouse contain metadata describing each sample retrieved from GEO with GEOquery. The files are then deployed to Amazon S3 and made accessible for download. The 3D visualization of all samples on the ARCHS4 website is calculated with t-SNE^43^ after quantile normalization and log2 transformation of the human and mouse samples separately. The t-SNE procedure uses a perplexity of 50 for the sample centric embedding, and a perplexity of 30 for the gene-centric embedding using the Rtsne package in R^44^. The integration of the processed data into GEO series landing pages is achieved through the ARCHS4 Chrome extension. The extension, freely available at the Chrome Web Store, first detects whether a GEO GSE landing page is currently open in the browser. It then requests the matching GSE series matrices from ARCHS4 containing the gene expression counts and metadata information for the GSE. Additionally, the ARCHS4 Chrome extension requests JSON objects with pre-computed clustered gene expression for visualizing the samples with Clustergrammer^31^. Summary statistics of sample counts and tissue-specific samples are saved in a dedicated database table to be accessed by the ARCHS4 website landing page for display.

### Sample search with reduced dimensionality

To enable reliable similarity search of signatures within the ARCHS4 data matrix, the matrix is compressed into a lower dimensional representation. A projection that maintains pairwise distances and correlations between samples is computed with the JL method^21^. The JL-transform reduces the original gene expression matrix $E \in N \times M$ where *N* is the number of genes and *M* is the number samples, into a matrix $\hat E \in S \times M$, with $S < N$. A subspace of 1000 dimensions captures the original correlation structure with a correlation coefficient of 0.99 (Fig. 3a). For implementing the ARCHS4 signature search, a projection matrix $D_{\rm JL} \in 1000 \times N$ is used to calculate $\hat E = D_{\rm JL} \times E$. The human and mouse matrices are handled separately. For user queries, input signatures $\vec s = [s_1,s_2, \ldots ,s_n]$ are projected onto a lower dimension $\widehat {\vec s} = D_{\rm JL} \times \vec s$. Since ${\mathrm{cov}}(\widehat {\vec s},\hat E) \approx {\mathrm{cov}}(\vec s,E)$, this method enables responsive real-time signature similarity search with low error (Fig. 3b).

### The ARCHS4 interactive website

The front-end of ARCHS4 is hosted on a web server derived from a tutum/lamp Docker image that is pulled from Docker Hub. It is a web service stack running on a UNIX-based operating system with an Apache HTTP server and a MySQL database. ARCHS4 is an AJAX application implemented with PHP and JavaScript. All visual data representations are implemented in JavaScript. The sample statistics overview of the landing page is implemented using D3.JS^45^. On the data view page, the sample and gene three-dimensional embedding is visualized using Three.js and WebGL^46,^ which enable the responsive visualization of thousands of data points in 3D. Data-driven queries such as signature similarity searches are performed in an R environment hosted on a dedicated Dockerized Rook web server. On start-up, the Rook server automatically retrieves all necessary data files, including the JT-transformed gene expression table, as well as the transformation matrix, and loads them into memory for fast access from an S3 cloud repository. All Docker containers can be load-balanced and run on a Mesosphere computer cluster with redundant hardware. The load balancing and port mapping is controlled through a HAProxy service. The MySQL database is hosted as an RDS Amazon web service.

### Prediction of biological function and protein interactions

Gene–gene co-expression correlations across all human genes can be utilized to predict gene function and PPI by exploiting the fact that genes that co-express have the tendency to also share function and physically interact. First, expression matrices from ARCHS4 mouse, ARCHS4 human, GTEx^14^, and CCLE^47^ were organized into genes as the rows and samples as the columns. For the ARCHS4 data matrices, 10,000 samples were randomly selected to construct gene expression correlation matrices for mouse and human separately. For GTEx and CCLE, all available samples (9662 and 1037, respectively) were used to build the co-expression correlation matrix for all human genes. For ARCHS4 data, functional prediction accuracy increases with the number of samples included, while gains become marginal with more than 10,000 samples. Interestingly, even with a subset of 100 randomly selected samples, functional prediction accuracy is high. The quantile normalization function from the Bioconductor package preprocessCore^48^ was used to normalize gene counts across samples. From the extracted expression matrices, all pairwise gene correlations were calculated. For each gene set $g_j \in {\rm GS}$ and each gene *g*_*i*_ the mean correlation of the genes in the gene set to *g*_*i*_ was calculated. Self-correlations when $g_i \in {\rm gs}_j$ were excluded. Hence, the resulting gene set membership prediction matrix ${\rm GM} \in M \times N$ for *M* genes and *N* gene sets is generated by the following procedure:

$${\rm GM}_{ij} = {\mathrm{mean}}\left( {{\mathrm{cor}}\left( {g_i,g_j} \right)} \right).$$

GM_*i*_ is then sorted from high to low based on correlation level. For each row *i* within GM, a vector $\vec s = [s_{1{\rm GS}1},s_{2{\rm GS}2}, \ldots ,s_{n{\rm GS}n}]$ is then constructed where $s_{n{\rm GS}n} \in \{ 0,1\}$ and $s_{j{\rm GS}j}$ is 1 if gene *g*_*i*_ is already known to be in the gene set GS. This vector is sorted and used to compute the AUC from the cumulative sum of $\vec s_i$ using trapezoidal integration. To predict PPI, the three PPI networks, hu.MAP^32^, BioGRID^33^, and BioPLEX^34,^ are first converted to a gene set library as described by Ma’ayan et al.^49^. Then, to predict PPIs, the same procedure as described for functional predictions was applied.

### Data availability

The ARCHS4 website is accessible at https://amp.pharm.mssm.edu/archs4. On the site all processed data is available at the Download tab. Source code for the project is provided at https://github.com/MaayanLab/archs4. Source code is available under the Apache Licence 2.0. Provided gene expression files are made available under the Creative Commons Attribution 4.0 International License (Creative Commons License). All data is free to use for non-commercial purposes.

## Electronic supplementary material

Peer Review File
