# Supplementary material for: Massive mining of publicly available RNA-seq data from human and mouse
Source: Nat Commun. 2018 Apr 10;9:1366. doi: 10.1038/s41467-018-03751-6 (PMC5893633; doi:10.1038/s41467-018-03751-6)
Supplement: Supplementary file 1 — Peer Review File [file 41467_2018_3751_MOESM1_ESM.pdf]

Editorial Note: This manuscript has been previously reviewed at another journal that is not operating a transparent peer review scheme. This document only contains reviewer comments and rebuttal letters for versions considered at Nature Communications.

REVIEWERS' COMMENTS:

Reviewer #1 (Remarks to the Author):

The authors have addressed all my major concerns, good job!

Reviewer #2 (Remarks to the Author):

The authors largely addressed my previous comments. I do think the comparisons with other efforts in the new table should be presented in a more fair/comparable fashion. This would involve adding a line that describes whether each approach/pipeline/effort was alignment-based or alignment-free. It seems clear, at least to me, that the cost saving is from using the alignment-free kallisto rather than performing alignments with STAR or Rail-RNA (as the authors did not provide a per/sample cost estimate even in the revision)

Editor comment: Please address the remaining points from Reviewer 2

Reply: We added a column to the table to indicate the whether alignment-free quantification was implemented by each of the resources we compared.
